# Food Targeting: Determination of the Cocoa Shell Content (*Theobroma cacao* L.) in Cocoa Products by LC-QqQ-MS/MS

**DOI:** 10.3390/metabo10030091

**Published:** 2020-03-05

**Authors:** Nicolas Cain, Christian Marji, Kristian von Wuthenau, Torben Segelke, Markus Fischer

**Affiliations:** Hamburg School of Food Science—Institute of Food Chemistry, University of Hamburg, Grindelallee 117, 20146 Hamburg, Germany; nicolas.cain@chemie.uni-hamburg.de (N.C.); christian.marji@chemie.uni-hamburg.de (C.M.); kristian.wuthenau@chemie.uni-hamburg.de (K.v.W.); Torben.Segelke@chemie.uni-hamburg.de (T.S.)

**Keywords:** metabolomics, metabolic profiling, LC-QqQ-MS/MS, validation, cocao, *Theobroma cacao* L., cocoa shell, chemometrics

## Abstract

A targeted metabolomics LC-ESI-QqQ-MS/MS application for the determination of cocoa shell based on 15 non-polar key metabolites was developed, validated according to recognized guidelines, and used to predict the cocoa shell content in various cocoa products. For the cocoa shell prediction, different PLSR models based on different cocoa shell calibration series were developed and their suitability and prediction quality were compared. By analysing samples from different origins and harvest years with known shell content, the prediction model could be confirmed. The predicted shell content could be verified with a deviation of about 1% cocoa shell. The presented method demonstrates the suitability of the targeted application of metabolomic profiling for the determination of cocoa shell and its applicability in routine analysis is discussed.

## 1. Introduction

The term cocoa is used for cocoa nibs (cotyledons) obtained from the seeds of the cocoa tree Theobroma cacao L. as well as for the processed products. The main cocoa bean-growing countries in the 2017/18 harvest year were in decreasing order: Côte d’Ivoire, Ghana, Indonesia, Ecuador, Cameroon, and Nigeria. Today, around 4,500,000 tons of cocoa beans are produced worldwide [1].

The processing of cocoa involves several steps starting with the harvesting of the ripe cocoa fruits. The ripe cocoa fruits are opened for fermentation and the cocoa seeds are layered together with the pulp in boxes or on banana leaves and covered. Fermentation takes place over a period of 2–8 days, depending on the variety and fermentation method [2,3]. After fermentation, the cocoa beans are subjected to a washing and cleaning step to remove pulp residues and adhesions. The water content of the cocoa beans must then be regressed. In order to reduce the water content to 5%–8%, where the transport conditions are stable and the beans will not get mouldy, the cocoa beans are dried in the sun, turning several times [4].

The dried cocoa beans are increasingly processed directly in growing countries into semi-finished products such as cocoa mass, cocoa powder and cocoa butter. In the 2013/14 harvest year, around 45% of the harvested beans were processed directly in the countries of origin [5].

To process the dried cocoa beans into semi-finished products, they are separated from impurities and roasted at temperatures of 130–150 °C for 15–45 min [6]. The roasting process is followed by the separation of the cocoa shell. For the separation, the cocoa beans are broken by rolling mills or by collision witch steel plates [7]. The cocoa shell is transmitted to vibrating machines, where the lighter shells can be separated due to the difference in density. A further separation takes place by an air stream [8]. In addition to the whole bean roasting, nib roasting or liquor roasting can also be carried out [9]. The roasted cocoa nibs, which in a controlled process contain only a technologically unavoidable proportion of shell, are used for the production of various cocoa products such as chocolates. The separated shell, which can be considered a by-product of cocoa processing, is used as animal feed or starting material for the extraction of theobromine [10].

### 1.1. Reasons for Cocoa Shell Detection

In the industrial processing of cocoa beans into semi-finished or finished cocoa-based products, the shell is separated from the nibs after fermentation and roasting. However, complete separation is not always possible for technological reasons. This is mainly due to the high diversity of the cocoa beans, which can vary greatly in size, shape and volume [11]. Depending on the growing region, variety and processing of the beans, cocoa masses produced from these beans can have a shell content of 11–17%, based on the fat-free dry matter [12].

A high percentage of cocoa shell can in many respects be considered a quality impairment. The insertion of cocoa shell can result in the transfer of substances that are hazardous to health (e.g., mycotoxins or heavy metals) to the processed product [13,14,15]. Furthermore, a high shell content can wear out machine parts due to abrasion and negative product properties (e.g., sandy mouth feel) [15,16].

In general, manufacturers or distributors of cocoa products must be able to verify the specifications of their products. Especially, because cocoa beans are increasingly being processed directly in the countries of harvest. This requires valid methods for quality assurance as well as methods for monitoring cocoa processing and, if necessary, for monitoring process optimization. This need was met here with a metabolomic profiling approach. Following this approach, a valid and powerful detection method was developed based on mass spectrometric detection of several different key metabolites.

### 1.2. Legal Basis for the Cocoa Shell Content

Already in 1918, the Cocoa Powder Order established a limit value of 5% for the cocoa shell content in cocoa powder in Great Britain [17]. This limit value and further definitions for different cocoa products were defined in 1973 in Directive 73/241/EEC. The content of cocoa shell and radicles may no longer exceed 5% of the non-fat dry matter in cocoa nibs. This Directive was revised and replaced by Directive 2000/36/EC, in which the legally binding limit value no longer applies. In accordance with previous legislation and the international food standards for cocoa liquor set by the Codex Alimentarius Commission, the same limit was recommended and the value was set in the cocoa processing industry [18]. A shell content of 5% is technologically unavoidable and corresponds to the general perception of the trade. In the case of cocoa products that exceed this technologically unavoidable content, it can be assumed that stretching, processing of inferior goods or inadequately controlled process management has taken place.

### 1.3. History of the Cocoa Shell Determination

Until today, various analytical approaches have been developed to determine the cocoa shell content in cocoa products. In 1899, the first methods for the cocoa shell detection were developed [19]. The methods developed were based on the microscopic analysis of cocoa samples, which is based on the detection of vascular bundles, mucous cells, or sclereids. However, the content of cocoa shells could only be estimated to a limited extent because the individual tissue elements cannot be clearly identified, especially in processed products [20,21].

Further early gravimetric analytical methods are based on the physical differences of the shell and cocoa content [22].

Detection of the cocoa shell content based on differing concentrations of ingredients, especially the higher ash content in the cocoa shell also failed because the concentration depends on the origin and processing stages of the cocoa samples [23].

Furthermore, a photometric method was developed which is still in use today. This method is known as the blue value method and is based on the derivatization of the fatty acid tryptamides into a blue complex [24]. The fatty acid tryptamides are contained in much higher concentration in the cocoa shell than in the cocoa nibs and can therefore be used as a marker. Although the method is officially recognized by the International Office of Cocoa, Chocolate and Sugar Confectionery [25], and it was shown that this method is very selective, the tryptamide content is subject to biological variance, which depends on the provenance of the cocoa samples [26]. Furthermore, this method can only be applied to cocoa butter. Other cocoa products such as cocoa powder or cocoa masses cannot be determined photometrically using this method due to the intensive inherent colouring.

In addition to the photometric determination of fatty acid tryptamides another method based on high-pressure liquid chromatographic separation and detection using a fluorescence detector was developed in 2001 [27]. Further analyses have shown that the fatty acid tryptamides analysed are not only found in the cocoa shell but also in the cocoa nibs. However, this method is also based solely on the detection of a few compounds, which may be subject to natural fluctuations due to the biological variability of the cocoa beans.

The latest detection experiment was performed using NIR [28]. The method was only developed for cocoa powder and only two different cocoa shell samples were used for the manufacturing of the samples, so the influence of cocoa samples with different origins, harvest years and varieties was not considered, although a strong dependence between these data and the composition of the cocoa shell could be demonstrated in previous works.

In a previous non-targeted metabolomics approach, key metabolites could be detected, whose concentration in the cocoa shell is many times higher than in the cocoa nibs. The following substance classes were used for the cocoa shell determination: fatty acid tryptamides, fatty acid serotonides (fatty acid 5-hydroxy-tryptamides), Ceramide derivatives, Tocopherol derivatives and triacylglycerols [29]. In other studies, 5-hydroxy-tryptamides were used for the detection of cocoa shell in addition to fatty acid tryptamides [30]. The concentration independence of these compounds with respect to different origins, roasting, fermentation, harvest years, and varieties has been verified by extensive studies. Based on these key metabolites, a targeted LC-ESI-QqQ-MS/MS method was developed in the present study.

The method used in this study differs from all other methods in the application of a larger number of different key metabolites from different substance classes. The main advantage is the better resolution of the fingerprint through the application of 15 different metabolites, whereas the established methods are exclusively based on the determination of one or few metabolites. 

## 2. Results and Discussion

### 2.1. Extraction Solvent Optimization

The best results were achieved with a mixture of 30% MTBE and 70% isopropanol. As a result of the optimization of the extraction solvent, chloroform was replaced by the much safer MTBE. MTBE has already been used in various studies as a very strong extraction agent [31,32]. The results and scores of the different extraction solvents are shown in the Supplementary Materials (Tables S1 and S2).

### 2.2. Validation

Validation of the targeted LC-ESI-MS/MS method was carried out for all available reference standards in accordance with guidelines for bioanalytical method validation of the FDA and the German DIN 32645. Validation was performed both with (matrix calibration) and without (base validation) the presence of cocoa matrix. Reference substances could not be purchased for all key metabolites, but it was ensured that at least one reference was acquired for each substance class, allowing their validation results to be extrapolated to all representatives of that class. The reference standards and assignment of the key metabolites are displayed in Table S3 in the Supplementary Materials.

The results of the basic and matrix calibration of the reference substances are listed in Table 1. A very high linearity with a correlation coefficient of >0.99 could be achieved for all standards. Dihydroceramide (d18:0/16:0) showed a linear range from 0.0002–1 µM. A smaller linear range could be detected for the other substances. The limit of detection was less than 1 nM for all compounds.

Furthermore, the values of accuracy and precision were calculated for all calibration points of the standards. The calculated values for both accuracy and precision at LLOQ (defined as the concentration of the lowest standard in the calibration series) were below the required 20% by the guidelines. Accuracy and precision were further verified at the medium and highest concentrations. All compounds were able to meet the required maximum deviation of 15% at both concentrations. In order to detect matrix effects, a matrix calibration was also performed. The same parameters were calculated as for the basic calibration. The results are shown in Table 2. Very high linearity with a correlation coefficient of >0.99 could also be achieved here for all standards. As a result of the matrix influence, a slightly smaller linear range was obtained, and the detection limit was also marginally increased. The requirements for accuracy and precision could also be met with the matrix calibration. The matrix has only a minor influence on the measurement.

### 2.3. Prediction Model

In order to quantify the cocoa shell in cocoa products, a prediction model for the cocoa shell was developed using a cocoa shell calibration series with defined shell contents. PLSR models were created for the prediction. The suitability of PLSR models for the prediction of cocoa shell content or other research topics has already been demonstrated in many papers [28,33,34]. Two different calibration series were prepared in order to compare and determine which method is best suited for preparation and prediction. In order to create a reliable and robust model, the investigated key metabolites have to show linearity in the relevant concentration range (0–10%) in order to be able to predict the concentration of the shell in cocoa products. The linearity of the key metabolites was already confirmed in a previous work for 17 of the 18 compounds using a LC-ESI-QTOF system. The preparation of the shell calibration series is described in more detail in the material and methods chapter.

To verify the suitability of the calibration series, eight samples of different origins with a known shell content were prepared and analysed. Furthermore, 15 different commercially acquired chocolates and a cocoa butter were analysed. In addition, a calibration series in the range of 0–100% cocoa shell was used to verify the linearity of the metabolites over the entire concentration range. The cocoa shell series were analysed in a fivefold determination and in randomized order.

When transferring the method from the LC-ESI-QToF- to the LC-ESI-QQQ-system, three of the 18 key metabolites could not be included in the targeted method. In addition to using a different analytical system, for the targeted approach was also used a different extraction solvent, which may have had an influence on the concentration of the key metabolites in the extracts. As a result of these changes, three of the metabolites no longer showed a linear relationship in the relevant concentration range.

The concentration of the 15 key metabolites was within the calculated linear ranges. Therefore, the evaluation and calculation of the regression models were performed based on the remaining 15 metabolites. In order to be able to understand the influence of the individual metabolites to the model, an evaluation based on linear regressions of each key metabolite was also carried out to predict the cocoa shell content. Coefficient of determination and regression equations of each key metabolite are shown in Table S4 in the Supplementary Materials. For every key metabolite a linear regression was calculated and the cocoa shell content of samples with known shell content was then individually predicted. For illustrative purposes, Table 3 presents the predicted cocoa shell content of samples from Ecuador and Ivory Coast.

The predicted shell content of the samples clearly showed that the calculated shell contents for each key metabolite scattered very strongly and influenced the value differently. The prediction of the sample from Ecuador varied between 1.10% and 22.41% and that of the sample from the Ivory Coast between −0.9% and 5.2%. However, the main metabolites do not always indicate high or low cocoa shell content in the different samples. The metabolites Hexacosanoic acid tryptamide and Heneicosylic acid serotonin can be considered as examples of this relationship. The prediction of the cocoa shell content by Hexacosanic acid tryptamide results in a significantly higher value for the sample from Ecuador than the average value and a significantly lower value for the sample from Ivory Coast. The opposite can be observed for Heneicosylic acid serotonin. Therefore, it is necessary to use as many metabolites as possible from different substance classes in order to obtain a robust model. By calculating the mean value, the actual content can almost be determined.

**Figure 1:**
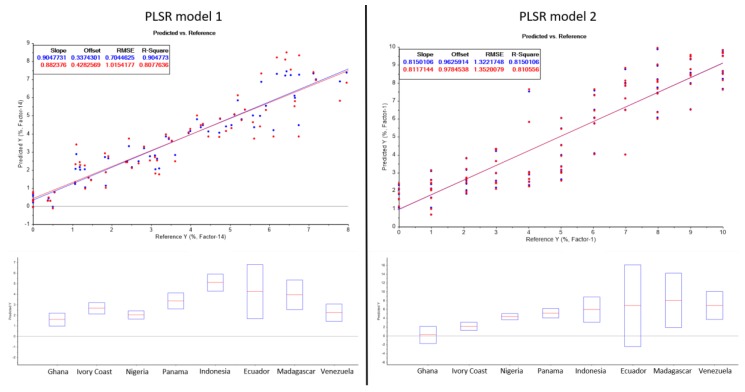
PLSR models of cocoa shell calibration series 1 and 2 and the results of the cocoa shell contents of 8 different samples from various origins calculated using the developed PLSR models. The parameters and results of the calculated PLSR models are shown in Table S5 in the Supplementary Materials.

Figure 1 shows the PLSR model of the first calibration series. The model was calculated by the Unscrambler X 10.3 software using all 15 key metabolites and carrying out a full cross validation. All variables were weighted equally, and a mean-centering was carried out with the data set. The model shows a linear dependence between the area of the metabolites and the cocoa shell content with a coefficient of determination of 0.9. The deviations of the samples with a higher cocoa shell concentration is greater than for the samples with a lower concentration. The greater deviation in cocoa samples with a higher shell content may be attributed to greater inhomogeneity in these samples.

The samples were prepared by mixing cocoa nibs and cocoa shell powder as shown in 2.2. The model should then be applied to samples with known cocoa shell content to verify the validity. The predicted cocoa shell contents of the samples are shown in Table 4. Overall, satisfactory results were achieved with this model. When predicting the shell content, 5 out of 8 samples showed suitable results with a very small deviation from the actual content. Samples from Ghana, Côte d’Ivoire, Nigeria, Panama and Indonesia showed only slight deviations to the actual concentrations. A deviation of 1% cocoa shell can be considered as a wholly satisfactory result. Samples from Madagascar and Venezuela contained more than 7% cocoa shell and showed very poor results with a deviation of 3.32% and 5.08% respectively. As already observed during the creation of the model, the deviations of the model in a higher concentration range were very big. This could explain the large deviations of the samples with a high shell content.

Furthermore, a PLSR model was calculated on the basis of the second calibration series. As with the first model, a linear dependence of the metabolites in the investigated concentration range of 1–10% is given. The coefficient of determination is 0.82. If the mean values of the calibration points are solely taken into account for the calculation of the PLSR model, a regression coefficient of 0.97 results. The same data pre-treatment and validation was performed as for the first calibration series. The dispersion of values in the higher concentration range from the first model does not apply to this model. As in the case of the first model, the samples with a known shell content were analysed. Comparing the predicted and the actual shell contents, there is only a small deviation for all samples. Unlike to the first model, suitable results are also obtained for the samples from Madagascar and Venezuela. As powders were used for the preparation of the samples, a certain inhomogeneity remains even when mixing. The potential inhomogeneity of the calibration series and of the produced samples with a defined shell content can cause the deviations of the calibration points and the deviations of the predicted results.

Whereas in the first model the cocoa shell and nibs were weighed in directly, in the second model cocoa shell and nibs mixtures were produced and a certain quantity was taken (the weighings of the samples are shown in the supporting information). For this reason, slightly different results can be expected between the two attempts. If the applicability and feasibility of the two dilution series are compared, it becomes clear that the second calibration series is much easier and time-saving to carry out. In addition, the deviations of the shell content in the samples with known shell content and in the chocolate samples are much smaller. Therefore, the second model is better suited for calibrating and determining the shell content in various cocoa samples.

After the predictive quality of the PLS models were confirmed by samples with known shell content, the model was also applied to samples with unknown shell content and other stages of processing. These samples included 14 different chocolates and three cocoa butters. Since the PLSR model of the second calibration series showed much better results over the whole concentration range, this model was used for the prediction of samples with unknown shell content. Fourteen chocolate samples were purchased from different manufacturers and consist of different chocolate variants, such as white, milk and dark chocolates. The predictions of the chocolates and cocoa butters are shown in Table 5. Besides the predicted cocoa shell content, the table also shows the cacao content and the calculated cocoa shell content in relation to the used cocoa products. The listed percentages of cocoa are the sum of cocoa mass and cocoa butter.

The shell content of the cocoa butters were predicted to be 6.79%. This result coincides with the assumption that the lipophilic key metabolites of the shell pass over to the cocoa butter when the cocoa butter is pressed. The white chocolates as well as the milk chocolate, showed the lowest proportion of cocoa shell. White chocolate has a cocoa butter content of approx. 28% [35]. Since the cocoa butter contains approx. 6.8% cocoa shell, accordingly the white chocolate should contain approx. 2% cocoa shell. This calculation corresponds to the obtained results. Milk chocolate has a cocoa mass content of approx. 12% and 18% cocoa butter. Though, low cocoa shell contents of approx. 2% are also expected here, which is in line with the obtained results. The greater the amount of cocoa, the higher the expected cocoa shell content should be. This correlation could also be observed for the analysed chocolates. An exception was the chocolate with a cocoa content of 99%. A lower cocoa shell content was detected here than in the chocolate of the same manufacturer with 85% cocoa. This could be explained, by better process control and the use of high-quality raw materials. When calculating the cocoa shell content of the individual chocolates in relation to the used cocoa, it is noticeable that, for most chocolates, cocoa with a cocoa shell content of 6 ± 2% was used. Only one chocolate showed a markedly higher cocoa shell content of over 12%. The increased content could be attributed to faulty production. However, since only one bar of chocolate was used for the analysis, this could also be an outlier. Furthermore, the cocoa shell content could also be non-homogeneously distributed in the chocolate, although the detection was carried out in triple determination and the individual results showed only a very small deviation.

**Figure 2:**
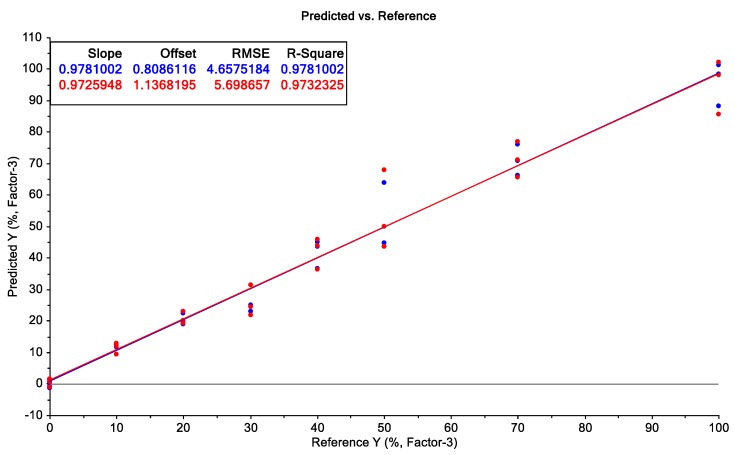
PLSR model of cocoa shell calibration series 3 (0–100% cocoa shell).

In addition to the calibration series between 0% and 10%, samples with a shell content between 0% and 100% were prepared and analysed to verify the linear dependence of the key metabolites outside the previously investigated concentration range. Figure 2 shows the linearity over the entire concentration range of 0–100% shell. Therefore, the metabolites can predict the shell content independently of the contained concentration. Cocoa shells are used as a by-product for theobromine extraction or as animal feed [10]. Since the key metabolites are linear up to 100% cocoa shells, the method can also be used to control cocoa shells.

In this study, a LC-ESI-QqQ-MS/MS targeted method for the determination of the cocoa shell content in different cocoa and chocolate products was successfully developed and validated. Besides the suitability for different cocoa products, the method is also applicable for samples of different origin. Furthermore, the method is characterized by a simple and fast implementation. By replacing chloroform with MTBE as an extraction solvent, the negative impact on the environment and users has been reduced. With the developed method, the cocoa shell content of different cocoa products can be predicted with an accuracy of approximately 1% cocoa shell. The accuracy of the prediction could be further increased by using reference substances for every key metabolite for external calibration and isotope-labelled standards for internal calibrations. External calibration using reference standards would solve both the inhomogeneity problem of the cocoa shell calibration series and the extensive time involved in producing and weighing the cocoa shell calibration series. Furthermore, calibration by means of external and internal standards could provide absolute quantitation of the key metabolites and the method could be transferred directly to commercial or industrial laboratories. The presented method can be considered as a supplement to the NIR detection method published in 2019 [28]. The NIR method can be regarded as a rapid screening method and should the analysis reveal a shell content close to the limit value, this can be reviewed using the presented method here. Furthermore, the NIR method is a non-targeted method and therefore the method does not provide the level of selectivity that is given by the LC-ESI-QqQ method. Using the NIR method impurities in the samples that also can cause bands at the selected wavelength could have an influence on the results of the method. Because of the multiple reaction monitoring method developed in this work, this influence of impurities can be avoided. However, the NIR method has only been applied to cocoa powder and not to cocoa masses or chocolates, while the suitability of the here presented method has already been confirmed for these matrices as well.

## 3. Material and Methods

### 3.1. Reagent and Chemicals

Ultrapure water was obtained by purifying demineralized water in a Direct-Q 3 UV-R system (Merck Millipore, Darmstadt, Germany). LC-MS grade isopropanol was purchased from Honeywell (Seelze, Germany), ammonium formate solution (10 M in water) were supplied by Sigma-Aldrich (Steinheim, Germany) and HPLC grade chloroform and Methyl tert-butyl ether (MTBE) was supplied by Carl Roth (Karlsruhe, Germany).

The reference standards DL-Tocopherol palmitate were purchased from Gerbu Biotechnik (Heidelberg, Germany), N-palmitoyl-D-erythro-shinganine (C16 Dihydroceramide (d18:0/16:0) from Avanti Polar Lipids (Alabaster, AL), Arachidonic acid serotonin from Cayman Chemical (Ann Arbor, MI) and Docosanoic acid tryptamide from Sigma-Aldrich (Munich, Germany).

### 3.2. Cacao Samples

Three different cocoa shell calibration series were produced. Firstly (A), cocoa shell and cocoa nibs were weighed directly together in a quantity of 50 mg in a concentration range between 0–7.5% of the shell. Cocoa beans from the Ivory Coast of the harvest year 2016/17 were used for this purpose. Secondly (B), a mixture was prepared by combining defined proportions of cocoa shell and cocoa nibs homogenates. For better miscibility and homogeneity, the cocoa shell homogenate was treated with a ball mill at 3.1 m/s for 5 min., while the cocoa nibs homogenate already had a very fine homogenous structure. Afterwards, the cocoa shell was mixed with cocoa nibs in various proportions and homogenized in a mortar with a pestle. A total of eleven different samples were prepared in a concentration range of 0–10% of cocoa shell (app. 5 g each). A mixture of cocoa beans from the Ivory Coast, Ecuodor, Ghana, Cameroon and Indonesia from the harvest years 2015–2017 was used for this purpose. Weighings and resulting cocoa shell contents of the calibration series are displayed in Table S6 to S9 in the Supplementary Materials.

Third (C), a calibration series in the concentration range from 0% to 100% cocoa shell was applied in the same way as the second approach to verify the linear relationship of the key metabolites in the higher concentration range.

To verify the quality of prediction for the shell content, 8 samples of different origin and with different cocoa shell contents were prepared analogously to (B). The composition of the samples is shown in Table 4.

Furthermore, 14 chocolates with different cocoa contents and 3 cocoa butter sample were analysed. Further information about the cocoa samples are shown in Table S10 in the Supplementary Materials.

### 3.3. Sample Treatment

Fermented cocoa beans were used for the calibration series and the other samples, which were treated as follows. Preparation of the samples included roasting, separation of the nibs from the shell and germ, homogenization and extraction. All samples were handled identically during all preparation and analytical steps. The sample material was stored at −80 °C until preparation.

The samples were first thawed at room temperature for one hour and afterwards roasted. Therefore, the beans were evenly distributed on a grid covered with aluminium foil and roasted at 145 °C in a drying oven for 30 min. After cooling the beans to room temperature, the cocoa nibs were separated from the shell and germ manually with the aid of a scalpel. In the following, the term “cocoa shell” represents both cocoa shell and germ. Subsequently, the cocoa nibs and the cocoa shell were homogenized separately. The homogenization was carried out with the addition of dry ice by means of a Grindomix GM 300 knife mill equipped with a stainless-steel grinding container and a full metal knife (Retsch, Haan, Germany). The homogenate was freeze-dried and stored at −80 °C until extraction and analysis. These homogenates were used for the production of the different mixture.

For the extraction 50 mg of cocoa nibs/shell samples were mixed with 1 mL of a cooled extraction solution (2-propanol/MTBE (7:3, *v/v*) and two steel balls were added. The Extraction was carried out by a Bead Ruptor 24 equipped with a 2 mL microtube carriage kit (Biolabproducts, Bebensee, Germany) at 3.1 m/s for 5 min. A further 1 mL of the extraction solution was added. After cell disruption, the extraction solution was centrifuged at 14,800 rpm for 5 min at 4 °C. The supernatant was taken up by means of disposable syringes, membrane filtered using a Rotilabo PTFE syringe filter, 0.45 µm pore diameter (Carl Roth, Karlsruhe, Germany), transferred to a vial and sealed with a crimp cap. Unless the analysis is done immediately after extraction, the samples were stored at −20 °C until measurement.

### 3.4. HPLC-ESI-QqQ-MS/MS Data Acquisition

The liquid chromatographic separation of the key metabolites was performed with the aid of a 50 mm × 2.1 mm i.d., 1.8 µm, ZORBAX RRHD HPLC column (Agilent, Waldbronn, Germany) and an Agilent 1200 series (Agilent, Waldbronn, Germany). The column temperature was set at 40 °C and the flow rate on 400 µL/min.

The mobile phase is composed of the solvent A water and B isopropanol/acetonitrile (3:2, *v/v*). 10 mM ammonium formate buffer with a pH of 3.5 was added to both eluents. The gradient elution was started at 70% B and kept constant for 2 min, in a second step linearly increased to 85% B in 2 min and afterwards to 100% B in 4 min. Further, 100% B was kept constant for 13 min and was moved back to 70% B in 0.1 min followed by 3.9 min of re-equilibration. The injection volume of each sample has been adjusted to 5 µL. The development of the method was based on the non-targeted method that was used to identify the key metabolites [29].

For quantitation an ESI-QqQ-MS/MS API 4000 system (Applied Biosystems, Darmstadt, Germany) equipped with a turbo ion spray source was used. The following source settings were used: Ion mode: positive; ion spray voltage: 5500 V; temperature: 450 °C; ion source gas 1: 30 psi; ion source gas 2: 70 psi; curtain gas: 20 psi; collision gas: 5 psi; ion spray probe position: vertical 3 and horizontal 5. For each metabolite two mass transitions were used. One was acquired for quantitation purposes (QNT: quantifier) and one for confirmatory purposes (QAL: qualifier). Dwell time was set to 20 ms for quantifier and qualifier.

For the optimization of the multiple reaction monitoring method (MRM), the optimal compound-dependent device voltages for each metabolite were verified by the automatic compound optimization of the Analyst Software (AB Sciex, version 1.6.3, Foster City, CA, USA). For optimization, a 10 μM solution of the reference standards was injected into the ion source with a flow rate of 10 μL/min. For metabolites for which no standard substance was available, the acquisition parameters were extrapolated from analogous substances of the same substance class. During the optimization process, the optimal declustering potential, entrance potential, collision energy and collision cell exit potential were identified for each substance regard to the largest signals. The acquisition parameters for each mass transition is presented in Table S11 in the Supplementary Materials. In order to counteract surrounding impacts and device-related influences, the samples were analysed in a randomized sequence and one in ten samples was measured a blank (extraction solvent).

### 3.5. Data Processing and Chemometrics

The integration was carried out with the Analyst software. Data pretreatment and multivariate statistics were performed by The Unscrambler X 10.3 software (Camo Software, Oslo, Norway). Prior to statistical processing, a mean centering of the data was calculated. For the prediction of the cocoa shell content, different partial least squares regression models (PLSR) were calculated based on the results of the cocoa shell dilution series. The cocoa shell content was predicted in samples of known and unknown cocoa shell concentrations using these models.

### 3.6. Method Validation

The validation was carried out to verify and determine linearity, limit of detection (LOD), precision, and accuracy according to accepted guidelines of the FDA [36] and DIN 32645 [37].

Since standards were not available for all compounds, the results of the reference standards of a substance class were applied to all key metabolites of this class. The transfer of the results to all compounds of the substance class was facilitated due to the very similar structure and fragmentation pattern. However, the results should only be regarded as an approximation, as there may be potential differences in the results due to different retention times and the slightly different structures.

Linearity was evaluated for the reference standards using a 20-point calibration curve in a fivefold determination. The range of the calibration curve is shown in Table 1. Linear range was calculated by means of Mandel’s fitting test [38]. Precision and accuracy were obtained determining the coefficient of variation at three concentration levels of the calibration curve. The calculated values for precision and accuracy should be within 15% of the nominal value. At the smallest concentration the value should not deviate by >20%. The limit of detection was calculated by the analysis of 10 independent samples of pure extraction solvent. The standard deviation of the signals and the slope of the calibration curve were used for the calculation.

### 3.7. Extraction Solvent Optimization

An extraction solvent optimization was performed to determine the solvent composition with the best extraction capacity. In addition, the extraction should be as simple as possible and contain a few intermediate steps in order to minimize loss of analyte and ensure fast performance. In the non-targeted studies, the extraction of the metabolites was performed with a mixture of isopropanol and chloroform (4/1, *v/v*), which showed the best results for the nonpolar metabolome of cocoa [29]. However, the use of chloroform is problematic because chloroform is suspected of causing cancer and poses a serious threat to the environment [39].

For the replacement of chloroform, an alternative extraction solvent should be found, which, due to its polarity, is capable of extracting the nonpolar metabolites. The extraction capacities of twelve different solvent combinations were tested in a triple setup. Different mixtures of methanol, acetonitrile and MTBE together with isopropanol were analysed. A cocoa shell sample was used as matrix. For the evaluation of the optimal extraction solvent, the mean value of the detected signal areas of the triple determination of the key metabolites was calculated for each extraction solvent composition and sorted by extraction capacity (signal area) in descending order. The extraction solvent composition with the highest signal area represents the best extraction capacity and received the rating one. Analogously, the extraction agent composition with the second highest signal area received the rating two and so on. Finally, the sum of the extraction solvent scores of all key metabolites was used as the basis for evaluating the extraction capability. The lower the sum of the scores of an extraction solvent composition, the higher the extraction capacity in relation to the key metabolites. After a mixture of MTBE and isopropanol had been identified as the optimal extraction solvent, the optimal ratio of the solvents was determined in a second approach.

## Figures and Tables

**Table 1:** Selected validation parameters for base and matrix calibration.

Reference Standard	Base Calibration				Matrix Calibration			
	Regression Equation	R^2^	Linear Range (µM)	LOD (nM)	Regression Equation	R^2^	Linear Range (µM)	LOD (nM)
Arachidonic acid serotonin	y = 248220.2 · x + 1533.5	0.999	0.001–1	0.09	y = 278210.6 · x − 986.1	0.999	0.002–0.8	0.08
Docosanoic acid tryptamide	y = 12455742.5 · x + 543249.3	0.992	0.06–1	0.82	y = 14596705.2 · x + 9745880.4	0.996	0.008–0.8	0.70
Dihydroceramide (d18:0/16:0)	y = 1773104.3 · x + 13728.51	0.998	0.0002–1	0.14	y = 1977769.7 · x − 39526.9	0.999	0.0004–0.8	0.12
16:0(2S-OH) Ceramide	y = 1782136.1 · x + 1191.2	0.999	0.001–1	0.31	y = 3298578.8 · x + 15185.9	0.999	0.002–0.8	0.17
α-Tocopheryl palmitate	y = 16762174.8 · x + 5161.2	0.998	0.0002–0.01	0.04	y = 352568.9 · x + 14953.1	0.992	0.008–0.8	1.87

**Table 2:** Calculated accuracy and precision of the base and matrix calibration at three different concentration levels.

Reference Standard	Calibration Level	Base Calibration		Matrix Calibration	
		Accuracy (%)	Precision (%)	Accuracy (%)	Precision (%)
Arachidonic acid serotonin	0.001 µM	12.85	11.65	8.72	8.83
	0.04 µM	9.42	3.78	6.67	4.68
	1 µM	0.45	2.14	1.34	14.11
Docosanoic acid tryptamide	0.06 µM	2.77	7.09	10.21	3.40
	0.2 µM	9.00	3.75	5.10	2.33
	1 µM	3.19	2.71	2.77	3.54
Dihydroceramide (d18:0/16:0)	0.0002 µM	5.66	11.31	6.58	4.67
	0.01 µM	8.75	8.91	4.78	5.43
	1 µM	1.11	3.39	2.26	4.31
16:0(2S-OH) Ceramide	0.001 µM	12.49	15.35	6.61	6.71
	0.01 µM	7.29	7.41	3.05	4.65
	1 µM	2.78	5.98	2.05	0.73
α-Tocopheryl palmitate	0.0002 µM	14.42	5.20	17.34	5.16
	0.001 µM	2.34	5.31	11.08	8.14
	0.01 µM	13.70	6.60	5.53	5.93

**Table 3:** Predicted cocoa shell content by linear regression of two different cocoa samples.

Key Metabolite	Calculated Cocoa Shell Content Ecuador Sample (%)	Calculated Cocoa Shell Content Ivory Coast Sample (%)
α-Tocomonoenol	9.0	1.7
Heneicosylic acid serotonin	0.9	2.0
Docosanoic acid serotonin	1.4	1.8
Lignoceric acid serotonin	4.1	1.0
Pentacosanoic acid serotonin	4.0	0.2
Hexacosanic acid serotonin	11	−0.8
Behenic acid tryptamide	4.5	1.7
Heneicosylic acid tryptamide	3.6	1.7
Tricosanoic acid tryptamide	6.1	1.8
Pentacosanoic acid tryptamide	14	1.0
Hexacosanic acid tryptamide	22	0.1
Dihydroceramide (d18:0/16:0)	5.5	5.2
Cer(d25:0(OH)/18:0(3OH))	5.1	2.4
α-Tocopherolpalmitat	4.3	0.0
Lignoceric acid tryptamide	8.7	1.2
Ø Average	6.9	1.4
Actual value	6.1	2.0

**Table 4:** Predicted and given cocoa shell content of 8 samples of different origins.

Sample	Shell Content (%)	Predicted Shell Content (Prediction Model 1) (%)	Predicted Shell Content (Prediction Model 2) (%)	Variance (Prediction Model 1) (%)	Variance (Prediction Model 2) (%)
Ghana	1.08	1.58	0.22	0.50	0.86
Ivory Coast	2.01	2.67	2.16	0.66	0.15
Nigeria	2.98	2.02	4.35	0.96	1.37
Panama	4.10	3.36	5.14	0.74	1.04
Indonesia	5.02	5.10	5.94	0.08	0.92
Ecuador	6.06	4.24	6.83	1.82	0.77
Madagascar	7.26	3.94	8.04	3.32	0.78
Venezuela	7.32	2.24	6.87	5.08	0.45

**Table 5:** Predicted cocoa shell content of different analysed chocolates and cocoa butters.

Sample	Predicted Shell Content (%)	Percentage of Cocoa (%)	Calculated Shell Content in Utilized Cocoa (%)
Milk chocolate	2.01	~30	6.70
White chocolate	2.78	~30	9.27
White chocolate	2.22	~30	7.40
Dark chocolate	4.12	55	7.49
Dark chocolate	4.75	50	9.50
Extra dark chocolate	5.36	73	7.34
chocolate (1)	4.13	33	12.52
chocolate (1)	4.23	55	7.69
chocolate (1)	5.35	66	8.11
chocolate (1)	6.70	88	7.61
chocolate (2)	2.37	50	4.74
chocolate (2)	3.01	70	4.30
chocolate (2)	5.74	85	6.75
chocolate (2)	5.00	99	5.05
Cocoa butters	6.79	100	6.79

(X) Manufacturers of Chocolate.

## Supplementary Materials

The following are available online at https://www.mdpi.com/2218-1989/10/3/91/s1, Table S1: Results of the extraction solvent optimization, Table S2: Results of the determination of the optimal MTBE and isopropanol composition, Table S3: Reference standards and assignment of the key metabolites, Table S4: Regression equations of the calibration series 2, Table S5: Parameters and results of the calculated PLSR models, Table S6: Weighings and resulting cocoa shell contents of the calibration series 1, Table S7: Weighings and resulting cocoa shell contents of the calibration series 2, Table S8: Weighings and resulting cocoa shell contents of the calibration series 3, Table S9: Weighings and resulting cocoa shell contents of samples with known cocoa shell content, Table S10: Cocoa and chocolate samples, Table S11: MRM transitions and acquisition parameters.

## Abbreviations Used

PLSR, Partial Least Squared Regression; EEC, European Economic Community; MTBE, Methyl Tert-Butyl Ether; MRM, Multiple Reaction Monitoring.
