# Supplementary material for: Food Targeting: Determination of the Cocoa Shell Content (Theobroma cacao L.) in Cocoa Products by LC-QqQ-MS/MS
Source: Metabolites. 2020 Mar 5;10(3):91. doi: 10.3390/metabo10030091 (PMC7143241; doi:10.3390/metabo10030091)
Supplement: Supplementary file 1 [file metabolites-10-00091-s001.pdf]

Table S1: Results of the extraction solvent optimization.

Extraction solvent	Isopropanol [%]	MTBE [%]	Acetonitril [%]	Methanol [%]	Sum of scores
1	80	20	0	0	46
2	50	50	0	0	37
3	20	80	0	0	74
4	80	0	20	0	75
5	50	0	50	0	79
6	80	0	0	20	105
7	50	0	0	50	117

Table S2: Results of the determination of the optimal MTBE and isopropanol composition.

Extraction solvent	MTBE [%]	Isopropanol [%]	Sum of scores
1	30	70	36
2	40	60	57
3	50	50	53
4	60	40	53
5	70	30	65
6	80	20	80

Table S3: Reference standards and assignment of the key metabolites.

Substance class	Reference standards	Key metabolites
Fatty acid tryptamides	Docosanoic acid tryptamide	Docosanoic acid tryptamide<br>Heneicosylic acid tryptamide<br>Tricosanoic acid tryptamide<br>Pentacosanoic acid tryptamide<br>Hexacosanic acid tryptamide<br>Lignoceric acid tryptamide
Fatty acid serotonins	Arachidonic acid serotonin	Heneicosylic acid serotonin<br>Docosanoic acid serotonin<br>Lignoceric acid serotonin<br>Pentacosanoic acid serotonin<br>Hexacosanic acid serotonin
Ceramide	Dihydroceramide (d18:0/16:0)	Dihydroceramide (d18:0/16:0)
	16:0(2S-OH) Ceramide	Cer(d25:0(OH)/18:0(3OH))
Tocopherol-Derivate	α-Tocopheryl palmitate	α-Tocomonoenol<br>α-Tocopheryl palmitate

Table S4: Regression equations of the calibration series 2.

Key metabolite	Regression equations	R2
α-Tocomonoenol	y = 4805.2 · x + 7684.8	0.978
Heneicosylic acid serotonin	y = 1667.9 · x + 5794.5	0.984
Docosanoic acid serotonin	y = 53050.7 · x + 214106.5	0.961
Lignoceric acid serotonin	y = 79445.3 · x + 414380.6	0.958
Pentacosanoic acid serotonin	y = 3281.0 · x + 14449.13	0.963
Hexacosanic acid serotonin	y = 6733.8 · x + 67877.8	0.900
Docosanoic acid tryptamide	y = 713457.1 · x + 3800340.5	0.973
Heneicosylic acid tryptamide	y = 9699.8 · x + 34095.5	0.970
Tricosanoic acid tryptamide	y = 32807.8 · x + 128215.6	0.971
Pentacosanoic acid tryptamide	y = 44778.0 · x + 257361.8	0.932
Hexacosanic acid tryptamide	y = 97658.2 · x + 735776.3	0.860
Dihydroceramide (d18:0/16:0)	y = 9566.1 · x + 13204.8	0.977
Cer(d25:0(OH)/18:0(3OH))	y = 4204.4 · x + 1279.4	0.982
α-Tocopherolpalmitate	y = 361.0 · x + 592.1	0.925
Lignoceric acid tryptamide	y = 927564.8 · x + 6667329.9	0.958

Table S5: Parameters and results of the calculated PLSR models.

PLSR model	Algorithm	Calibration				Cross-validation			
		RMSEC	R^2	Slope	Offset	RMSEC	R^2	Slope	Offset
Series 1	NIPALS	0.704	0.910	0.905	0.337	1.015	0.808	0.882	0.428
Series 2	NIPALS	1.322	0.815	0.815	0.963	1.352	0.811	0.812	0.978
Series 3	NIPALS	4.658	0.978	0.978	0.809	5.700	0.973	0.973	1.137

Table S6: Weighings and resulting cocoa shell contents of the calibration series 1.

Sample	Shell [mg]	Nibs [mg]	Content [%]	Sample	Shell [mg]	Nibs [mg]	Content [%]	Sample	Shell [mg]	Nibs [mg]	Content [%]
1_1	0	51.17	0	6_1	1.45	54.15	2 68	11_1	2.45	47.17	5.19
1_2	0	50.62	0	6_2	1.45	51.47	2.82	11_2	2.42	47.37	5.11
1_3	0	49.81	0	6_3	1.15	48.11	2.39	11_3	2.54	48	5.29
1_4	0	49.14	0	6_4	1.22	48.6	2.51	11_4	2.38	50.06	4.75
1_5	0	50.92	0	6_5	1.43	48.75	2.93	11_5	2.62	49.19	5.33
2_1	0.19	48.6	0.39	7_1	1.44	48.46	2.97	12_1	2.69	46.7	5.76
2_2	0.27	50.25	0.54	7_2	1.51	48.87	3.09	12_2	2.76	47.68	5.79
2_3	0.18	49.17	0.37	7_3	1.56	48.69	3.20	12_3	2.92	49.38	5.91
2_4	0.25	49.51	0.50	7_4	1.54	49.65	3.10	12_4	2.65	47.22	5.61
2_5	0.22	49.92	0.44	7_5	1.57	49.86	3.15	12_5	2.58	47.97	5.38
3_1	0.53	49.61	1.07	8_1	1.69	48.05	3.52	13_1	2.65	47.53	5.58
3_2	0.55	49.97	1.10	8_2	1.66	48.28	3.44	13_2	2.93	47.32	6.19
3_3	0.55	50.85	1.08	8_3	1.64	48.69	3.37	13_3	3.07	46.92	6.54
3_4	0.58	49.04	1.18	8_4	1.92	48.14	3.99	13_4	2.87	47.03	6.10
3_5	0.65	54.84	1.19	8_5	1.79	49.62	3.61	13_5	3.18	47.15	6.74
4_1	0.94	51.01	1.84	9_1	1.89	47.81	3.95	14_1	3.22	48.5	6.64
4_2	0.72	49.08	1.47	9_2	2.07	49.81	4.16	14_2	3.08	47.93	6.43
4_3	0.65	49.5	1.31	9_3	2.07	48.62	4.26	14_3	3.35	46.65	7.18
4_4	0.71	50.21	1.41	9_4	2.06	47.77	4.31	14_4	3.07	46.28	6.63
4_5	0.64	48.57	1.32	9_5	1.97	47.97	4.11	14_5	3.25	48.15	6.75
5_1	0.93	50.23	1.85	10_1	2.43	51.4	4.73	15_1	3.72	46.73	7.96
5_2	1.2	49.29	2.43	10_2	2.44	48.52	5.03	15_2	3.79	47.8	7.93
5_3	0.92	50.57	1.82	10_3	2.11	47.41	4.45	15_3	3.59	56.07	6.40
5_4	0.94	49.11	1.91	10_4	2.26	49.69	4.55	15_4	3.43	48.27	7.11
5_5	1.15	48.78	2.36	10_5	2.35	47.97	4.90	15_5	3.64	46.74	7.79

Table S7: Weighings and resulting cocoa shell contents of the calibration series 2.

Sample	Shell [mg]	Nibs [mg]	Content [%]
0	0	5.000	0
1	0.050	4.950	1.00
2	0.104	4.902	2.08
3	0.150	4.852	3.00
4	0.201	4.803	4.02
5	0.251	4.749	5.01
6	0.302	4.702	6.04
7	0.350	4.652	6.99
8	0.400	4.605	7.98
9	0.450	4.552	9.00
10	0.500	4.499	10.00

Table S8: Weighings and resulting cocoa shell contents of the calibration series 3.

Sample	Shell [mg]	Nibs [mg]	Content [%]
0_1	0	50.10	0.00
0_2	0	50.21	0.00
0_3	0	50.05	0.00
1_1	4.90	44.84	9.85
1_2	5.45	44.24	10.97
1_3	4.78	44.73	9.65
2_1	11.41	40.75	21.88
2_2	9.90	40.37	19.69
2_3	11.13	41.16	21.29
3_1	16.12	34.76	31.68
3_2	14.70	35.91	29.05
3_3	13.87	37.87	26.81
4_1	21.00	32.00	39.62
4_2	20.88	33.37	38.49
4_3	20.30	30.07	40.30
5_1	25.87	29.65	46.60
5_2	26.60	26.90	49.72
5_3	26.34	28.15	48.34
6_1	35.67	14.12	71.64
6_2	35.40	14.71	70.64
6_3	35.09	16.80	67.62
7_1	49.50	0	100.00
7_2	51.30	0	100.00
7_3	50.51	0	100.00

Table S9: Weighings and resulting cocoa shell contents of samples with known cocoa shell content.

**Sample**	**Shell [mg]**	**Nibs [mg]**	**Content [%]**
Ghana	10.79	990.1	1.08
Elfenbeinküste	20.11	979.9	2.01
Nigeria	29.80	970.1	2.98
Panama	41.00	959.2	4.10
Indonesien	50.27	950.2	5.02
Ecuador	60.71	941.3	6.06
Madagaskar	73.16	934.5	7.26
Venezuela	82.03	1039.3	7.32

Table S10: Cocoa and chocolate samples.

<table>
<tr><td>Sample name</td><td>Origin</td><td>Harvest year</td><td>Cocoa shell content</td></tr>
<tr><td>Ghana</td><td>Ghana</td><td>2016</td><td>1,08</td></tr>
<tr><td>Ivory Coast</td><td>Ivory Coast</td><td>2016</td><td>2,01</td></tr>
<tr><td>Nigeria</td><td>Nigeria</td><td>2016</td><td>2,98</td></tr>
<tr><td>Panama</td><td>Panama</td><td>2016</td><td>4,10</td></tr>
<tr><td>Indonesia</td><td>Indonesia</td><td>2015</td><td>5,02</td></tr>
<tr><td>Ecuador</td><td>Ecuador</td><td>2014</td><td>6,06</td></tr>
<tr><td>Madagascar</td><td>Madagascar</td><td>2015</td><td>7,26</td></tr>
<tr><td>Venezuela</td><td>Venezuela</td><td>2016</td><td>7,32</td></tr>
<tr><td></td><td></td><td></td><td></td></tr>
<tr><td>Cocoa shell and nibs for the calibration series A</td><td>Ghana</td><td>2016</td><td>Table S7</td></tr>
<tr><td>Cocoa shell and nibs for the calibration series B</td><td>Ghana</td><td>2016</td><td>Table S8</td></tr>
<tr><td>Cocoa shell and nibs for the calibration series C</td><td>Ghana</td><td>2016</td><td>Table S9</td></tr>
<tr><td>Milk chocolate</td><td rowspan="15" colspan="3">unknown</td></tr>
<tr><td>White chocolate</td></tr>
<tr><td>White chocolate</td></tr>
<tr><td>Dark chocolate</td></tr>
<tr><td>Dark chocolate</td></tr>
<tr><td>Extra dark chocolate</td></tr>
<tr><td>chocolate (1)</td></tr>
<tr><td>chocolate (1)</td></tr>
<tr><td>chocolate (1)</td></tr>
<tr><td>chocolate (1)</td></tr>
<tr><td>chocolate (2)</td></tr>
<tr><td>chocolate (2)</td></tr>
<tr><td>chocolate (2)</td></tr>
<tr><td>chocolate (2)</td></tr>
<tr><td>Cocoa butters</td></tr>
</table>

(X) Manufacturers of Chocolate

Table S11: MRM transitions and acquisition parameters.

Key metabolites	Sum formula	Precursor (DA)	QNT (DA)	QAL (DA)	RT (min)	Adduct ion	Dwell time [msec]	DP [V]	EP [V]	CE [V]	CXP [V]
α-Tocomonoenol	C29H48O2	429.245	165.1	205.1	8.53	H+	20	81	10	10	29
Heneicosylic acid tryptamide	C31H52N2O	469.419	143.9	161.1	8.67	H+	20	106	10	10	39
Docosanoic acid tryptamide	C32H54N2O	483.227	143.9	161.1	9.02	H+	20	106	10	10	39
Heneicosylic acid serotonin	C31H52N2O2	485.410	160.0	159.6	7.83	H+	20	101	10	10	49
Tricosanoic acid tryptamide	C33H56N2O	497.447	143.9	161.1	9.35	H+	20	106	10	10	39
Docosanoic acid serotonin	C32H54N2O2	499.427	160.0	159.6	8.25	H+	20	101	10	10	49
Lignoceric acid tryptamide	C34H58N2O	511.462	143.9	161.1	9.65	H+	20	106	10	10	39
Pentacosanoic acid tryptamide	C35H60N2O	525.457	143.9	161.1	9.96	H+	20	106	10	10	39
Lignoceric acid serotonin	C34H58N2O2	527.457	160.0	159.6	8.99	H+	20	101	10	10	49
Hexacosanic acid tryptamide	C36H62N2O	539.490	143.9	161.1	10.2	H+	20	106	10	10	39
Dihydroceramide (d18:0/16:0)	C34H69NO3	540.359	522.5	284.2	10.5	H+	20	71	10	10	29
Pentacosanoic acid serotonin	C35H60N2O2	541.472	160.0	159.6	9.32	H+	20	101	10	10	49
Hexacosanic acid serotonin	C36H62N2O2	555.488	160.0	159.6	9.63	H+	20	101	10	10	49
α-Tocopheryl palmitate	C45H80O3	686.444	669.6	149.2	13.9	NH4+	20	211	10	10	23
Cer(d25:0(OH)/18:0(3OH))	C43H85NO5	696.650	678.6	280.3	11.8	H+	20	71	10	10	17

*DP: declustering potential; EP: entrance potential; CE: collision energy; CXP: collision cell exit potential
